# Biological correlates of altered circadian rhythms, autonomic functions and sleep problems in autism spectrum disorder

**DOI:** 10.1186/s12991-022-00390-6

**Published:** 2022-05-09

**Authors:** Liliana Dell’Osso, Leonardo Massoni, Simone Battaglini, Ivan Mirko Cremone, Claudia Carmassi, Barbara Carpita

**Affiliations:** Department of Clinical and Experimental Medicine, University of Pisa, Via Roma 67, Pisa, Italy

**Keywords:** Autism spectrum disorder (ASD), Sleep disorders, Circadian rhythms, Melatonin, Autonomic nervous system

## Abstract

Autism spectrum disorder (ASD) is a neurodevelopmental condition characterized by a complex and multifaceted neurobehavioral syndrome. In the last decades, several studies highlighted an increased prevalence of sleep problems in ASD, which would be associated with autonomic system and circadian rhythm disruption. The present review aimed to summarize the available literature about sleep problems in ASD subjects and about the possible biological factors implicated in circadian rhythm and autonomic system deregulation in this population, as well as possible therapeutic approaches. Shared biological underpinnings between ASD symptoms and altered circadian rhythms/autonomic functions are also discussed. Studies on sleep showed how ASD subjects typically report more problems regarding insufficient sleep time, bedtime resistance and reduced sleep pressure. A link between sleep difficulties and irritability, deficits in social skills and behavioral problems was also highlighted. Among the mechanisms implicated, alteration in genes related to circadian rhythms, such as *CLOCK* genes, and in melatonin levels were reported. ASD subjects also showed altered hypothalamic pituitary adrenal (HPA) axis and autonomic functions, generally with a tendency towards hyperarousal and hyper sympathetic state. Intriguingly, some of these biological alterations in ASD individuals were not associated only with sleep problems but also with more autism-specific clusters of symptoms, such as communication impairment or repetitive behaviors Although among the available treatments melatonin showed promising results, pharmacological studies for sleep problems in ASD need to follow more standardized protocols to reach more repeatable and reliable results. Further research should investigate the issue of sleep problems in ASD in a broader perspective, taking into account shared pathophysiological mechanisms for core and associated symptoms of ASD.

## Introduction

The center of circadian rhythms regulation can be identified in the hypothalamus. It consists of several neuronal populations which produce neuropeptides and neurotransmitters with a crucial role for temperature, metabolic rate, thirst, hunger, sexual behavior, reproduction and emotional responses. The hypothalamus exerts its functions mainly through the productions of neuropeptides, such as the transcription factor orthopedia (OTP), crucial for proper development of diencephalic dopaminergic neurons or the steroidogenic factor 1 (SF-1), which is important for neuronal and structural connectivity. Another transcriptional factor, Sim-1, is implicated in hypothalamus differentiation [1]. Deregulation of autonomic system and of daily activity rhythms often precede underlying pathophysiological changes in the brain of subjects with neurodegenerative diseases, such as Alzheimer disease. Similarly, dysautonomias have been seen in metabolic syndromes, cardiovascular diseases, diabetes and cancer. In the last decades, several studies highlighted the importance of altered circadian rhythms, autonomic functions and sleep quality in ASD, while some authors reported associations between these alterations and specific autistic-like symptoms. Intriguingly, an abnormal connectivity between hypothalamus and amygdala has been associated with inappropriate responses to socially relevant stimuli in autism spectrum disorder (ASD) [2]. In particular, a dysfunction of the endogenous circadian system was hypothesized to be part of the neurodevelopmental alteration at the basis of the autistic condition [3]. Sleep problems are frequent among ASD individuals. Several hypotheses have been proposed for explaining this association, including a deficit in melatonin production, anxiety and arousal at the time of going to bed or specific gene mutations. Sleep disturbances have been related to the severity of ASD symptoms (restrictive and repetitive behaviors, difficulties in reciprocal and social interactions, irritability, etc.). It was recently reported an association between actigraphy-derived sleep efficiency, number of awakenings, anxiety and hyperactivity in ASD children. Other studies showed instead a positive correlation between sleep latency, affective problems and aggressive behavior in this population [4]. Some of the genes responsible of regulating circadian rhythms seem to be mutated or down-regulated in ASD, thus providing a molecular explanation for sleep problems: in this framework, deepening the knowledge about genes implicated in circadian rhythms deregulation could be crucial for the development of eventual biomarkers of sleep functions [5]. Circadian hormones such as melatonin and cortisol were also found to be altered in ASD. ASD individuals seem to show an altered production of melatonin, which, in turn, has been related to sleep difficulties, altered gastrointestinal motility, deficits in behavioral and emotional regulation, sensory protein dysfunction. As in the case of gene deregulation, alterations of melatonin/serotonin pathways could be promising biomarkers for ASD [6]. The increased cortisol levels of ASD people were related to hyperarousal and wake after sleep onset (WASO) duration in subjects with insomnia and in controls. On the other hand, a lower cortisol response was linked to poorer self-reported sleep-in healthy population [7]. Autonomic variables such as cardio-vagal activity and pupil size were also investigated in ASD, highlighting in ASD children a lower cardio-vagal activity as measured by heart rate variability (HRV) and increased sympathetic activity measured by means of urinary vanillylmandelic acid (VMA) [8]. It was pointed out that dysautonomias in ASD may be linked to the presence of repetitive behaviors [9]. Starting from these considerations, two important therapeutic strategies, such as melatonin supplementation and Early Start Denver Model (ESDM), a developmental and behavioral intervention, have been studied in ASD not only for sleep problems but also for improvements in social communications, stereotyped behaviors, rigidity, and anxiety [10]. In this review, we aimed to summarize previous reports about sleep problems in ASD children and adults, and about possible biological factors implicated in the alteration of circadian rhythms and autonomic function in this population, together with possible therapeutic approaches. Eventual shared biological underpinnings between ASD symptoms and altered circadian rhythms/autonomic functions will also be discussed.

## Methods

To identify the studies focused on the relationship between ASD and sleep problems, a comprehensive literature review was performed using multiple databases (Pubmed, Web of Science, Scopus). Key words used were “autism spectrum disorder”, “autism”, “neurodevelopmental disorder”, “sleep problem”, “sleep disorder”, “circadian rhythm”, “autonomic nervous system”, “pharmacological therapy”, “pharmacological treatment”, “biological correlates”, “hormone”. Articles were considered eligible if written in English from 1980 to 2022.

## Sleep problems in ASD: from childhood to adulthood

Several studies highlighted a link between ASD and sleep problems, reporting specific correlations with some symptoms. Besides questionnaires, methods used for evaluating sleep in children and adolescents with ASD include polysomnography and actigraphy. The first one measures multiple physiological sleep parameters, such as brain waves, eye movements, etc. However, in ASD children, due to sensory sensitivity related problem, systematic desensitization is often required. The actigraph is a portable accelerometer device that can be worn on the wrist or ankle and records different sleep variables such as time of sleep onset, total sleep time, morning waking time and frequency of night-time waking [11]. Actigraphy enables to distinguish ASD poor sleepers from good sleepers, because the first ones would show prolonged initial sleep latency, decreased sleep efficiency and increased number of night awakenings [12].

Noticeably, some studies also highlighted an association between sleep problems and other symptoms in this population. Considering children, Schreck et al. [13] reported a relationship between reduced sleep time and deficits in social skills in this population. Moreover, Sikora et al. [14] found that sleep problems were often associated with both externalizing (such as impulsivity and aggression) and internalizing symptoms (such as anxiety and depression). In a study conducted on hospitalized ASD children, higher scores on the Aberrant Behavior Checklist-Community scale (ABC-C) at admission were associated with fewer minutes slept during the last five nights of hospitalization. Meanwhile, sleep problems seem not to be influenced by the subtype of ASD, or by the degree of cognitive impairment. Goldman et al. [15] compared sleep problems of older children and adolescents with ASD with those of control toddlers and younger children. Participants were divided in four groups: less than 5 years, from 5 to 7, from 7 to 11, more than 11 years. While all groups reported sleep related problems, differences in the kind of problem were reported depending on age: younger children showed greater levels of sleep anxiety, bedtime resistance, parasomnias and night walking, while older children and adolescents more frequently reported short sleep duration, delays in sleep onset and daytime sleepiness [12, 15]. A recent study evaluated the association between sleep problems and autistic traits in toddlers (*n* = 426). The classification was based on the total Modified Checklist for Autism in toddlers (M-CHAT-JV) scores. The autistic group (*n* = 26) was composed mainly by males. In terms of sleep problems, three items were more frequently endorsed in the autistic group: “bedtime resistance” (time attended before going to bed), “abnormality in circadian rhythm” (the total sleep time varies from one night to another), “sleepiness outside the naptime”. It has emerged that children aged 3–5 years with more difficult temperaments showed substantially more bedtime resistance than children with less difficult temperaments. Bedtime resistance in children at around 18 months of age may be related to subclinical autistic traits. It has been suggested that children with ASD have a deregulation of sleep homeostasis, resulting in a reduction of sleep pressure [16]. Stressful events, such as home confinement during COVID-19 pandemic, were recently shown to exert an impact on sleep in ASD children. In particular, a study was conducted among Turkish ASD children (*n* = 46). Children were given different questionnaires, such as the children’s chronotype questionnaire (CCQ), autism behavior checklist (AuBC) and children’s sleep habits questionnaire (CSHQ). Results reported that ASD children had greater sleep problems and altered chronotype during home confinement period than during normal state. The severity of ASD symptoms increased together with the chronotype score and sleep score during home confinement. Interestingly, the level of sleep problems was considered a mediating factor in the relationship between CCQ score and autism symptom severity. Moreover, a significant correlation was found between increased CSHQ and CCQ score and ASD symptoms during the home confinement period [17]. Other authors also hypothesized that neonatal-irritable sleep–wake rhythm may be involved in ASD pathogenesis. Miike et al. [18] conducted an analysis among 177 ASD children and 203 controls. In the ASD group irritable/over-reactive types of sleep–wake rhythms were observed more frequently than in control group. These alterations may cause difficulties in raising the child, due to frequent waking up, difficulties in falling asleep, short sleep hours, continuous crying and grumpiness. Noticeably, the number of mothers who went to bed after midnight during pregnancy was higher in ASD group than in controls. Yavuz-Kodat et al. [19] investigated 52 ASD children aged 3–10 years. Sleep and circadian rest-activity rhythms were evaluated objectively with actigraphy and subjectively with the CSHQ, while the ABC-C was used for assessing behavioral difficulties. A shorter continuous sleep period was reported among children with high irritability compared to those with lower irritability. A similar trend emerged in children with high stereotyped behaviors compared to those with less stereotypies.

Other studies focused instead on ASD adolescents and adults, finding that insomnia, parasomnias and circadian rhythms disorders were common also in this population. Ballester et al. [20] led a study on 41 adults with ASD and intellectual disability (ID), and 51 typically developed adults. They used an ambulatory circadian monitoring recording wrist temperature, motor activity, body position, sleep and light intensity. ASD individuals showed several sleep difficulties, such as low sleep efficiency, prolonged sleep latency, increased number and length of night awakenings, with daily sedentary behavior and increased nocturnal activity. ASD adults had an advanced sleep–wake phase disorder. Sleep and markers of the circadian system were significantly different between adults with both ASD and ID and the control group. Sleep disturbances described for adults with ASD and ID are similar to those for ASD adults without ID. Even elderly ASD individuals seem to report more difficulties to fall asleep than other people. Jovevska et al. [21] enrolled in their studies subjects aged from 15 to 80 years (297 ASD participants and 233 controls). Sleep quality, sleep onset latency, total night sleep and sleep efficiency, as measured by Pittsburgh Sleep Quality Index, were analyzed. Moreover, the authors investigated autistic traits, mental health condition, medication, employment and sex as predictors of sleep quality. Results highlighted lower sleep quality and increased sleep onset latency in the whole ASD group rather than in the control one. ASD subjects also showed poorer sleep quality and longer sleep onset latency than controls of similar age, especially in adulthood and middle age, while no significant difference was found in adolescent and elderly ASD groups with respect to age-matched controls. Predictors seemed to account for the 21% of sleep quality variance in the ASD group, with sex as the strongest predictor (female sex more related to sleep problems). In the comparison group, the strongest predictor was mental health condition, with predictors accounting for 25% of sleep quality variance [21].

Some authors evaluated in adult samples the relationship between sleep problems and other features. Baker et al. [22] investigated sleep–wake rhythms in 36 ASD adults. Participants were assessed through a 14-day sleep–wake diary and 14-day actigraphy. ASD individuals seemed to show sleep patterns associated with circadian rhythm disturbances, although authors highlighted that the role of employment status, comorbid anxiety and depression should be taken into account. Another study from the same group evaluated employment status in ASD adults with sleep problems. A total of 72 individuals were enrolled in the study (36 ASD people, 36 controls). Subjects were assessed by questionnaires and 14-day actigraphy. 20 ASD individuals versus 4 controls met criteria for insomnia and/or circadian rhythm sleep–wake disorders. Adults with ASD who met the criteria for sleep disorders according to the International classification of Sleep-Disorders, Third edition, reported higher scores on the Pittsburgh Sleep Quality Index (indicating worse sleep quality) and were more frequently unemployed when compared with ASD adults without sleep problems. This result highlighted an association between sleep problems and unemployment [22]. Deserno et al. [23] also evaluated the impact of sleep on quality of life among 598 ASD adults. Autistic features, comorbid complaints, daily functioning and socio-demographic characteristics were evaluated by means of questionnaires. Results reported that sleep problems were an important predictor of subjective quality of life, while subjective experience of individual contribution in the society seemed to statistically predict the level of daily activities. The authors stressed how sleep problems may be considered an important treatment target for improving quality of life in ASD individuals.

In conclusion, while the literature seems to have reached a global agreement on the presence of sleep alterations in ASD subjects, during both childhood and adult life, results about the possible relationship between sleep problems and ASD symptoms are more limited and controversial, also due to high heterogeneity in the methodology and in the specific symptoms measured (see Table 1).

**Table 1:** Main findings about sleep problems in ASD

References	Participants	Materials	Main findings	Limitations and strengths
Schreck et al. [13]	ASD children (*N* = 55, range 5–12 years, mean age = 8.2 years)	A database of parent report of sleep problems of children based on a self-report demographic form, the GARS, and the BEDS	Sleep problems (fewer hours of sleep per night, increased sensitivity to environmental stimuli in the bedroom) and the diagnostic characteristics of autism (social skill deficits, communication problems, developmental sequence) disturbances) may be related	Limitations: absence of a control group of healthy individuals
Sikora et al. [14]	ASD children (*N* = 1193, *M* = 1014, *F* = 179, range = 4–10 years)	Measures included Children’s Sleep Habits Questionnaire, Vineland Adaptive Behavior Scales, Survey Interview Form, Second Edition, and Child Behavior Checklist	Even if sleep is negative related with internalizing and externalizing behaviour, it could be differently related with the acquisition of adaptive skills	Limitations: few differences were clinical significant; information about sleep and daytime behaviour was registered through parents reports; use of quartile scores as the cutoff between mild and moderate to severe sleep problems may not be valid; even though the study highlights the bidirectional relationship between sleep and daytime behaviour, other factors are supposed to be implicated; disproportion between male and female (*M*/*F* = 1014/179) Strengths: large sample size
Goldman et al. [15]	ASD children (*N* = 1859, range 3–18 years, mean age 80.1 ± 42.3 months)	CSHQ, PCQ	Sleep problems persist through adolescence in ASD with differences in types of problems experienced	Limitations: data are subjective and based on parental report; the cross-sectional nature of these data limits evaluation of a temporal relationship between age and sleep; the CSHQ and PCQ have not been validated with adolescents; not differentiation between males and females Strengths: large sample size
Ballester et al. [20]	ASD adults (*N* = 41, *M* = 31, *F* = 10, mean age = 33 ± 6 years) Typically developing adults (*N* = 51, *M* = 21, *F* = 30, mean age = 33 ± 5 years)	ACM recording wrist temperature, motor activity, body position, sleep, and light intensity	Poorer sleep conditions in adults with autism (increased sleep latency and number/length of night awakenings) resulted in decreased sleep efficiency	Limitations: these problems are life-long conditions, not only childhood related; parents or legal guardians of adults with ASD and ID with disturbed sleep were more likely to participate compared to those without sleep problems; ACM had not been validated in ASD to study sleep; while the comparison group was healthy, ASD one was medicated with polypharmacy; disproportion in *M*/*F* ratio particularly in the first group Strengths: it is a controlled study with similar numerosity between groups
Baker et al. [22]	ASD adults (*N* = 36, *M* = 17, *F* = 19, IQ > 80, range = 21–44 years, 20 of them met criteria for insomnia and/or a circadian rhythm sleep–wake disorder) and age and sex-matched controls (*N* = 36, *M* = 17, *F* = 19 4 of them met criteria for the disorders above)	14-day actigraphy assessment and questionnaire battery	It has emerged, for the first, time that sleep problems are associated with unemployment in adults with autism spectrum disorder	Strengths: it is a controlled study with similar *M*/*F* ratio in both groups. Limitations: studies with larger ASD samples would be useful
Deserno et al. [23]	ASD adults with subjective QoL as outcome (*N* = 598, *M* = 310, *F* = 288, range = 17–83 years) ASD adults with objective QoL as outcome (*N* = 544, *M* = 270, *F* = 274, range = 17–82 years)	For objective QoL: five subscales of the Autism Quotient (Baron-Cohen, Wheelwright, Skinner, Martin, & Clubley, 2001), five subscales of the Sensory Perception Quotient (Tavassoli, Hoekstra, & Baron-Cohen, 2014), seven items of the Insomnia Severity Index (Bastien, Vallières, & Morin, 2001). For subjective QoL: an item assessing how satisfied participants were with their own life	Sleep problems are highly influential in predicting long-term QoL in ASD individuals	Limitations: a specific set of symptom data and environmental factors was used for determination of multivariate pathways; individuals have a long-term level of happiness to which they always spontaneously return after life events of either valence; the authors were limited by online survey context and were unable to verify diagnosis and IQ of participants; the study sample has a large age range (17–83); there is not a control group Strengths: similar groups composition in terms of age and M/F ratio
Horiuchi et al. [16]	Autistic toddlers (*N* = 26, *M* = 20, *F* = 6) and non-autistic toddlers (*N* = 400, *M* = 184, *F* = 216) Total range:17–19 months Total mean age: 18 months	Japanese version of the M-CHAT-JV and the CASC	Autistic traits are associated with sleep problems in toddlers. As a result, daytime sleepiness might be a visible symptom that enables the earlier detection of ASD in children	Limitations: parents of toddlers who attended nursery school (199/426) had few information about their children’s daytime behavior; it is difficult to assess sleepiness in toddlers; few data are available on the reliability and validity of the CASC, and the previous studies that have used the CASC with toddlers; some items are not suitable for toddlers; disproportion between ASD and control group and in *M*/*F* ratio in ASD toddlers Strengths: controlled study
Türkoğlu et al. [17]	ASD drug-naïve children (*N* = 46, *M* = 38, *F* = 8, range: 4–17 years, mean age 7.89)	AuBC, CSHQ, CCQ	Children with ASD during the home confinement reported higher chronotype scores and autism symptom scores compared to the normal non-hone confinement state. The sleep problems of the children with ASD during the home confinement period mediated the relationship between chronotype score and severity of autism symptoms	Limitations: lack of a control group; small sample size; family members were not screened for psychopathology, post-confinement follow-up with participants was not done; disproportioned *M*/*F* ratio
Miike et al. [18]	ASD children from K-Development Support Center for Children (K-ASD, *N* = 121, *M* = 94, *F* = 27), ASD children from H-Children’s Sleep and Development Medical Research Center (H-ASD, *N* = 56, *M* = 40, *F* = 16), Children from recruited from four nursery schools in T-city (control) (*N* = 203, *M* = 104, *F* = 99)	Questionnaires to assess parent(s) of children with ASD and controls investigating: maternal lifestyle during pregnancy, neonatal sleep patterns, status of parent(s) in the child-rearing years	Neonatal sleep–wake rhythm abnormalities, especially in irritable-type neonates, are important precursors for future ASD development and so it is important to pay much more attention to the maternal role in fetal chronobiology formation and to circadian rhythm formation	Limitations: disproportioned *M*/*F* ratio in ASD groups Strengths: it is a controlled study
Yavuz-Kodat et al. [19]	ASD children (*N* = 52, *M* = 41, *F* = 11, range = 2.75–9.57 years, mean age = 5.39 ± 1.50 years)	Sleep and circadian rest–activity rhythms were objectively measured with actigraphy and subjectively with the Children’s Sleep Habits Questionnaire. Behavioral difficulties were assessed using the ABC-C	Problem behaviors were strongly accounted for by both sleep and circadian rhythm disturbances. Particularly, the longest continuous sleep episode is a novel clinically meaningful sleep parameter to consider, especially in children with severe sleep disorders	Assessment of both sleep and circadian rhythms with actigraphy is a strength because it is an objective measure, but also it is a limitation due to the fact that it remains a proxy of the circadian timing system. Another limitation: the lack of a control group
Jovevska et al. [21]	ASD group (*N* = 297, mean age = 34.36 ± 15.24) and comparison group (*N* = 233, mean age = 33.01 ± 15.53)	PSQI to examine sleep quality, SoL, total night sleep, and sleep efficiency. Other predictors of sleep quality: autistic traits, mental health condition, medication, employment, and sex	Autistic adolescents and adults, particularly females, remain vulnerable to sleep problems. Times where the risk is highest are early and middle adulthood	Limitations: sleep was measured using a self-report, retrospective questionnaire; the study was cross-sectional and so long-term trends for sleep quality in the context of chronicity or aging are not captured; autistic adults with intellectual disability were not included; about half the autistic sample was female, but the generally accepted male:female ratio in autism spectrum disorder is 4:1; the sample consisted of volunteers who responded to an advertisement, and not all participants had responses for all variables Strengths: a large part of ASD population included (18–80 years old); large sample size, including an age-matched comparison group; the first study which examines male–female differences in sleep in autistic adolescents and adults

*ASD* autism spectrum disorder, *GARS* Gilliam autism rating scale, *BEDS* behavior evaluation of disorders of sleep, *CSHQ* children’s sleep habits questionnaire, *PCQ* parental concerns questionnaire, *ACM* ambulatory circadian monitoring, *ID* intellectual disability, *QoL* quality of life, *IQ* intelligence quotient, *M-CHAT-JV* Modified Checklist for Autism in Toddlers, *CASC* Child and Adolescent Sleep Checklist, *AuBC* autism behavior checklist, *CCQ* children’s chronotype questionnaire, *PSQI* Pittsburgh Sleep Quality Index, *SoL* sleep onset latency

## Biochemical correlates of altered circadian regulation in ASD

### Genetic pathways

Several genes have been related to alterations of circadian rhythms. Among them, *CLOCK* gene was reported to be constitutive in the central nervous system (CNS). It was highlighted that the regulation of the cellular circadian clock would be the result of a particular loop including the transcriptional activators CLOCK and BMAL1, which stimulate the expression of *cryptochrome (Cry)* and *period (Per)* genes. The protein product of the genes, after having formed dimers in the cytoplasm represses their own transcription, via the inhibition of *CLOCK-BMAL1*, while on the other hand they activate *BMAL1*, by an unknown mechanism. As a result, they contribute to the constitution of a mammalian cell-autonomous oscillator. *CLOCK* genes are expressed not only in CNS, but also peripherally, in the oral mucosa, skin and blood. The co-expression of the gene in the blood, hippocampus and prefrontal cortex allow hypothesizing that its presence peripherally could be a good biomarker of the gene expression in the brain [24]. The importance of gene *CLOCK* and its epigenetic modulation in the circadian rhythmicity also emerges from evidences of hypo-methylation of *CLOCK* promoter, as well as of a higher *CLOCK* expression in blood cells of nightshift workers [25] *CLOCK*’s products play an important role in the dopaminergic outputs regulation, a pathway linked to many psychiatric disorders. CLOCK protein controls tyrosine hydroxylase (TH), cholecystokinin (CCK) and many other regulators of dopaminergic transmission. Meanwhile, acetylation of CLOCK is thought to regulate cortisol signaling responses thanks to both the reduction in the binding of glucocorticoid receptors to glucocorticoid response elements and the transcription repression of many glucocorticoid-responsive genes related to hypothalamic–pituitary–adrenal axis [26]. While circadian genes in ASD could be highly polymorphic, a dysfunction of circadian genes may be implied in ASD pathogenesis, or may contribute to its pathophysiology. In this framework, it is worth noting that a decreased expression of CLOCK seems to be present in individuals with ASD and some studies examined the specific role of CLOCK genes in this population. Period circadian regulator 1 (PER1), a CLOCK gene expressed in brain areas which affect sleep–wake transition, was also suggested to be mutated in ASD subjects. This alteration was hypothesized to be involved in the oscillations of the circadian clock in ASD, eventually also by affecting melatonin release [27]. Starting from the consideration that neuroanatomical and cellular changes potentially related to social impairment are particularly evident in the medial prefrontal cortex (mPFC) among ASD individuals, a study investigated the mPFC impaired molecular networks in a valproic acid (VPA) rat model of ASD. They found in the mPFC of this rat model 2 subsets of genes with a different expression: one was involved in circadian rhythm regulation, while the other featured collagen genes acting within the extracellular matrix [28].

Some human studies are also available in this field. Hu et al. [5] enrolled a group of ASD individuals who were divided on the basis of scores reported in the Autism Diagnostic Interview-Revised (ADIR) in three phenotypic groups. Their lymphoblastoid cell lines (LCL) were analyzed by means of microarray analyses, comparing the clinical groups with healthy controls. The authors identified a set of differentially expressed genes in each ASD group as well as a set of genes altered in all ASD groups. In the ASD group associated with higher severity, 15 genes regulating circadian rhythm were reported to be differentially expressed with respect to other groups, including the gene encoding for aralkylamine *N*-acetyltransferase (AANAT), an enzyme which catalyzes the first step of the biochemical conversion of 5-HT to melatonin, a key regulator hormone of circadian cycle. Noticeably, these genes were also linked to metabolic and neurological alteration typical of ASD. Among the genes reported to show an altered expression in all ASD groups, 20 novel genes associated with androgen sensitivity were revealed, leading authors to hypothesize that testosterone levels may be a risk factor for ASD [5]. More recently, a Japanese study investigated the mutations in *CLOCK* and related genes among ASD individuals versus controls. Many genetic variants located in the *CLOCK* gene locus, such as *rs3762836*, were found exclusively in ASD patients with sleep disorders [26, 29]. In a study on 28 ASD individuals and 23 controls, the coding regions of 18 canonical CLOCK genes and CLOCK-controlled genes were sequenced, reporting that mutations of circadian-relevant genes able to affect gene function were more frequent among ASD subjects than among controls [29]. Finally, a more recent work by Abel et al. [30] investigated the issue from another perspective, searching for possible genetic and biological mechanisms involved in the increased rate of sleep problems in this population. They found several overlapping genes between ASD and sleep problem gene sets, including CACNA1C gene, which is implicated in the regulation of calcium channels and has a circadian expression pattern, supporting circadian entrainment.

It should be noted that research in this field focused on different gene sets, with a lack of confirmation studies: thus it did not allow reaching a proper understanding, nor clarifying whether the association between ASD and altered gene expression related to circadian rhythms would be specific or possibly shared also by other mental disorders. In addition, very limited evidence is available on the association between gene variants and the specific presence of sleep problems in this population. In conclusion, while reports of altered CLOCK gene expression in ASD may suggest possible genetic underpinnings for circadian rhythmicity problems in these subjects, results in this field should be considered as preliminary (see Table 2).

**Table 2:** Genetic alterations in circadian rhythm of ASD individuals

References	Participants	Materials	Principal findings	Strengths and limitations
Nicholas et al. [27]	ASD probands and all their parents (*N* = 90, *M* = 65, *F* = 25 for the first stage of the study; *N* = 20, *M* = 14, *F* = 6 for the second stage of the study)	For screening candidate genes genotyping SNPs	Significant association (*P* < 0.05) for two single-nucleotide polymorphisms in *per1* and two in *npas2*; in *npas2* 40 out of the 136 possible two-marker combinations were significant at the *P* < 0.05 level. The best result was between markers rs1811399 and rs2117714, *P* = 0.001. In *per1* the significant result was for the markers rs2253820–rs885747. Epistatic clock genes may be involved in the etiology of autistic disorder. Problems in sleep, memory and timing are all characteristics of autistic disorder and aspects of sleep, memory and timing are each clock-gene-regulated in other species	Limitations: lack of a control group and the higher number of males compared to females
Hu et al. [5]	Three groups of autistic probands, selected after the exclusion of females, individuals with cognitive impairment, genetic or chromosomal abnormalities, born prematurely and comorbid psychiatric disorder and a control group (non-autistic controls)	DNA microarray analyses	In the most severely affected ASD group, 15 genes, which regulate circadian rhythm, have neurological and metabolic functions deregulated in ASD, were found. From other groups, 20 genes were pointed out, mostly located in non-coding regions and associated with androgen sensitivity	Limitations: the lack of the exact number of participants in each group and information about age and gender of the included people; epigenetic modifications related to inflammatory status Strengths: utility of subdividing individuals with ASD on the basis of cluster analyses of ADIR scores that incorporate all three core domains of ASD (as described in the accompanying manuscript)
Yang et al. [29]	ASD patients (*N* = 28, 14 of them with sleep problems and 14 without sleep problems. In the first group, *M* = 5 and *F* = 9, with an age range 3–28 years, while in the second group *M* = 12 and *F* = 2, with an age range 3–19 years) Healthy controls (*N* = 23)	Sequencing of the coding regions of 18 canonical clock genes and clock-controlled genes; direct sequence analyses verified detected mutations and additional control individuals were screened	Mutations in circadian-relevant genes affecting gene function are more frequent in patients with ASD than in controls. Circadian-relevant genes may be involved in the psychopathology of ASD	Limitations: small sample size Strength: presence of a control group (even if it is not known its internal composition)
Olde-Loohuis et al. [28]	Wistar rats	Rat mPFC collection, RNA isolation, RNA sequencing, gene ontology analysis, cDNA synthesis, qRT-PCR	Three different subsets of genes discovered: the first involved in the regulation of circadian rhythm, the second contributing to extracellular matrix, the third important to understand autism at a molecular level	Limitations: it is a study conducted on animals model, and it is difficult to make precise distinction between groups Strengths: it suggests a possible linking between circadian rhythm and molecular basis of autism

*ASD* autism spectrum disorder, *SNPs* single-nucleotide polymorphisms, *ADIR* autism diagnostic interview-revised, *mPFC* medial prefrontal cortex, *qRT-PCR* qReal time-polymerase chain reaction

### Role of melatonin

Melatonin is a circadian hormone which crosses the placental barrier and exerts a crucial role in fetal neurodevelopment. It is implicated in circadian physiology, and specifically in the sleep wake and core body temperature rhythm. It is secreted by pineal gland cells thanks to synchronization of these cells with 24-h day/night cycle made by photosensitive ganglion cells of the retina, which perceive environmental light [31]. Melatonin is produced by pineal gland during the night, starting from serotonin (5-HT). The conversion of 5-HT in melatonin features two enzymatic steps and the intermediate *N*-acetylserotonin (NAS) [32]. From a pharmacokinetic point of view, melatonin is subject to hepatic metabolism: its poor adsorption and substantial hepatic first pass metabolism explain the low bioavailability of this molecule. Melatonin is also metabolized in 6-sulfatoxy-melatonin and then excreted in the urine [33–35]. While a low amplitude and a possible delayed melatonin rhythm have been associated with increased sleep problems, melatonin deregulation has been supposed to be one of the main responsible for alteration of circadian rhythms in ASD. On the other hand, aberrant phase cycle of melatonin has been related to seizures and electroencephalogram (EEG) discrepancies in ASD individuals [3].

In a systematic review, Rossignol et al. [36] summarized results from 35 studies about melatonin in ASD. The authors concluded that levels of melatonin or melatonin derivatives were below average in individuals with ASD compared with controls. Melatonin pathway was reported to be altered in several ASD subjects, seeming also to correlate with ASD symptoms. In particular, abnormalities in genes involved in melatonin production and receptor function were reported, while some studies highlighted lower melatonin or melatonin metabolite concentrations, as well as altered melatonin circadian rhythm in ASD individuals when compared with healthy subjects or laboratory reference ranges. However, other studies reported instead daytime melatonin levels significantly higher in ASD than in controls [36]. On the basis of previously reported alterations of melatonin and 5-HT (hyperserotonemia) in ASD patients, more recently Pagan et al. [32] proposed that disruption of 5-HT-NAS-melatonin pathway may be a promising biomarker for ASD. To confirm their hypothesis, whole-blood 5-HT, platelets NAS and plasma melatonin were assessed in 278 patients with ASD, 506 first-degree relatives, 416 sex- and age-matched controls. Results confirmed the presence of hyperserotonemia and the deficit in melatonin production in the ASD group with respect to controls, together with increased platelet NAS levels. Relatives’ group reported intermediate levels between ASD and control groups. Interestingly, a strong correlation was found between platelet NAS and plasma melatonin levels in patients with ASD (more than in relatives’ group and controls), probably due to a common factor of deregulation. The melatonin deficit was significantly associated with insomnia. Other studies also focused on relatives of ASD patients, such as the study of Braam et al. [33], which found a significant lower 6-SM excretion rates in mothers of ASD children compared to controls [34, 35].

As reported above, some of the works reviewed by Rossignol et al. [36] investigated genes coding for melatonin receptors such as melatonin receptor 1A (MNTR1A), MTNR1B, GPR50, reporting, in a case, variants in genes MTNR1A and MTNR1B among 2.8% of ASD individuals [37]. Moreover, authors that investigated enzymes involved in hormone synthesis, such as Acetylserotonin *O*-methyltransferase (ASMT), showed a single-nucleotide polymorphisms in 2.6% of ASD individuals compared with healthy controls [38], while in another study two single-nucleotide polymorphisms in ASMT (rs4446909 and rs5989681) were reported to be more frequent among ASD individuals and were associated with a decrease in ASMT enzymatic activity and lower plasma melatonin levels [39]. Several other studies focused on genetic mutations of factors implied in the melatonin pathway in ASD. Wang et al. [40] also showed new coding mutations of ASMT in 6 ASD individuals, after having investigated the neighboring region in 398 subjects with ASD and in 437 controls. Veatch et al. [41] highlighted in ASD children treated with melatonin an association between sleep onset delay and dysfunctional variants in genes related to melatonin pathway, such as cytochrome (CYP) 1A2. The children who responded to treatment with melatonin showed a strong correlation between their genotypes in ASMT and CYP1A2. Another study focused on the genetic pathway of melatonin deregulation: they enrolled 295 ASD individuals, 362 European controls, and 284 subjects from human genome pluriethnic diversity panel, investigating the presence of rare variants of MTNR1A and MTNR1B. Their findings revealed MTNR1A and MTNR1B mutations linked to an alteration of the receptor functional properties: a significant association was detected between ASD and two variants in affected males [42, 43]. In ASD samples a reduction in pineal gland volume (PGV) was also highlighted [44, 45]. This volume reduction seemed related to primary insomnia, parasomnias and problems waking in the morning [45]. Pagan et al. [44] analyzed post-mortem pineal glands, gut samples and blood platelets of 239 individuals, respectively, for melatonin and 5-HT production and found a reduction of two enzymes related to melatonin synthesis (AANAT and ASMT) in blood platelets from individuals with ASD. However, the disruption of melatonin synthesis was not sufficient to induce 5-HT accumulation. The authors reported a significant correlation between ASMT deficits in blood platelets of ASD people and insomnia. Melatonin, ASMT, AANAT were significantly decreased in ASD individuals when compared with controls. By analyzing gastrointestinal tract of ASD people, no difference was highlighted between ASD individuals and controls with respect to 5-HT content. Melatonin content was instead lower in ASD patients than in controls. NAS was increased in blood platelets of ASD patients who had a disruption of melatonin synthesis. These authors also investigated the presence of ASMT mutations, finding that, in pineal glands of ASD patients, both degradation and synthesis of ASMT were impaired. Some interesting alterations in melatonin pathway were also observed by the same authors in first-degree relatives of ASD individuals. In particular, ASMT activity was significantly reduced in platelets of ASD subjects’ first-degree relatives when compared with controls, even if AANAT activity resulted diminished only in ASD participants and not in the group of relatives. Maruani et al. [45] evaluated PGV with magnetic resonance imaging (MRI) and early morning melatonin levels in 215 individuals (78 subjects with ASD, 90 unaffected relatives, 47 controls). Results highlighted that plasma melatonin levels were significantly different depending on the group and on the PGV. In another study, exons of 7 ASD patients and 17 controls were sequenced for ASMT and two rare variants were identified. 12 out of 290 individuals with ASD carried ASMT micro-duplication, versus 10 out of 324 of controls.

It should be noted that, in ASD subjects, a deregulation of melatonin production was related to sleep difficulties, but also to altered gastrointestinal motility, deficit in behavioral and emotional regulation as well as to sensory protein dysfunction [6]. In this framework, a positive correlation was reported between levels of 6-SM, severity of verbal communication impairment and daytime sleepiness in ASD subjects [46], whereas another work showed an association between lower mean serum melatonin level and abnormal EEG in ASD people [47]. However, other authors did not found instead correlations between Autism Diagnostic Interview and lower ASMT [39].

Finally, Rossignol et al. [36] also reported studies where administration of melatonin to ASD subjects had a beneficial effect in total sleep duration, number of night-time awakenings and sleep onset latency compared to placebo. In particular, some of these studies highlighted in ASD individuals treated with melatonin an improvement in daytime behaviors, such as less behavioral rigidity, ease of management for parents and teacher, better social interaction, fewer temper tantrums, less irritability, more playfulness, better academic performance and increased alertness. In a low number of subjects, minor side-effects related to melatonin treatment were also reported, such as mild morning tiredness, headache, dizziness, but also diarrhea, night-time awakening and excitement before going to bed [36].

Despite the majority of studies in this field seem to have reached an agreement about the presence in ASD population of altered melatonin levels and metabolism, it should be considered that altered melatonin was reported to be associated in this field not only with sleep problems but also with other symptoms. From this perspective, further studies are needed for clarifying if melatonin alteration may be involved in sleep problems directly or indirectly among ASD subjects, and if it should be considered as a causative factor, a consequence or a parallel process with respect to the neurodevelopmental condition (see Table 3).

**Table 3:** Biological studies on melatonin in ASD

References	Participants	Materials	Main findings	Strengths and limitations
Rossignol et al. [36]	Initial studies review: 35 reviewed independently by two reviewers Five of them were investigated through meta-analysis	Database used: PubMed, Google Scholar, CINAHL, EMBASE, Scopus, ERIC Quality of studies was assessed through Downs and Black checklist	Nine studies measured melatonin or its metabolites in ASD: all reported at least one alteration (four studies: abnormal melatonin circadian rhythm; seven studies: below average physiological levels; four studies: positive correlation between melatonin/derivatives levels and autistic behaviors). Five studies reported gene abnormalities that could decrease melatonin production or impair melatonin receptor function in a small percentage of ASD children	Limitations of the review: small sample size; variation in protocols for measuring changes in sleep parameters; five studies contained a mixture of individuals with ASD and other developmental disabilities
Wang et al. [40]	ASD children (*N* = 398, *M* = 367, *F* = 31, range 2–17 years) and healthy controls (*N* = 437, *M* = 406, *F* = 31)	Genotyping sequences in ASMT, DNA analysis and prediction the effects of coding non-synonymous variants on protein function	Four rare ASMT mutations were found only in ASD group (p.R115W, p.V166I, p.V179G, and p.W257X)	Limitations: low sample size for a rare genetic mutations investigation; important disproportion between *F* and *M* composition (367/31 in ASD group vs. 406/31 in control one); lack of information on the clinical and biochemical impacts of the ASMT deleterious variants and/or SNPs; other genes in melatonin pathway were not sequenced Strengths: controlled study
Veatch et al. [41]	ASD individuals (*N* = 29, *M* = 24 and *F* = 5, 15 of them underwent analysis for ASMT sequences, while 14 of the total for CY1A2 genotypes)	Examination of variation in two melatonin pathway genes, ASMT and CYP1A2	Higher frequencies than currently reported for variants evidenced to decrease ASMT expression and related to decreased CYP1A2 enzyme activity; a relationship between genotypes in ASMT and CYP1A2 was revealed; expression of sleep onset delay relates to melatonin pathway genes	Limitations: lack of a well-defined control group; all 11 individuals who participated in the melatonin trial were responsive to treatment; minimalization of the environmental effect of poor sleep habits through parent sleep education
Pagan et al. [32]	Unrelated patients with ASD (*N* = 278), first-degree relatives (129 unaffected siblings, 377 patients) and controls (*N* = 416)	Serotonin, melatonin and the intermediate NAS measured through whole-blood serotonin, platelet NAS and plasma melatonin	In patients the melatonin deficit was only significantly associated with insomnia. Impairments of melatonin synthesis in ASD may be linked with decreased 14-3-3 proteins. Disruption of the serotonin-NAS-melatonin pathway is a very frequent trait in ASD patients and may be a useful biomarker for a large subgroup of these individuals	Limitations: not equally subdivision between three groups Strengths: two groups of possible controls (unaffected siblings and other controls)
Pagan et al. [44]	ASD patients (*N* = 239), ASD parents (*N* = 303), unaffected siblings (*N* = 78), controls (*N* = 278)	Examination of melatonin synthesis in post-mortem pineal gland, serotonin synthesis in gut samples, blood platelets	Melatonin deficits in ASD depends on reduction activity of both enzymes implicated in melatonin synthesis (AANAT and ASMT)	Limitations: small number of patient samples Strengths: three different groups
Braam et al. [33]	Mothers of an ASD child (*N* = 60, mean age: 42.9 ± 5.7 years), control group of mothers (*N* = 15, mean age = 44.3 ± 9.7 years)	6-SM concentration	6-SM levels were significantly lower in mothers with an ASD child than in controls, and so, low parental melatonin levels could be one of the contributors to ASD and possibly ID etiology	Limitations: differences between number composition of both groups and small sample size; CYP1A2 activity not measured; different children ASD etiologies Strength: presence of a control group
Maruani et al. [45]	ASD individuals (*N* = 81), unaffected relatives (*N* = 90), control participants (*N* = 48)	PGV estimation based on magnetic resonance imaging; blood sampling and plasma melatonin measurement	Patients had both morning melatonin levels and PGV lower than controls; plasma melatonin was correlated to the group of the participant, but also to the PGV melatonin; variations in ASD could be mainly driven by melatonin pathway dysregulation	Limitations: melatonin was detected only in mornings; difficulties in exactly detecting PGV; numeric differences between the first two groups and the third Strengths: three experimental groups

*ASD* autism spectrum disorder, *6-SM* 6-sulphatoxy-melatonin, *ASMT* acetyl-serotonin-*O*-methyl-transferase, *AANAT* aryl-alkylamine-*N*-acetyltransferase, *MTNR1A* melatonin receptor 1A, *MTNR1B* melatonin receptor 1B, *GPR50*G protein-coupled receptor 50, *CYP1A2 *cytochrome P450 1A2, *NAS* *N*-acetyl-serotonin, *PGV* pineal gland volume

### Cortisol levels and hyperarousal

ASD children and adolescents often experience anxiety symptoms like “butterflies”, nausea or sweating, which may cause delays in sleep onset and even insomnia. ASD people have more pre-sleep arousal than typically developed individuals: the main worry is that of have difficulties in falling asleep. Pre-sleep arousal and anxiety were hypothesized to be involved in the difficulty in falling asleep among ASD children and adults [22]. In this framework, it should be noted that a deregulation of hypothalamic pituitary adrenal (HPA) axis and of cortisol production was reported among ASD subjects and it was linked to a deregulation of circadian rhythms [48]. However, while more literature is available about altered HPA in ASD, few studies specifically evaluated the association between these alterations and the actual presence of sleep problems.

Some of the works in this field focused on measuring cortisol/corticosteroids levels in different specimen. In an investigation led by Priya et al. [49], the urinary level of free cortisol, corticosteroids, VMA and 5-hydroxyindole acetic acid were determined among children with low functioning (LFA), medium functioning (MFA) and high functioning (HFA) ASD and in controls. Corticosteroids excretion levels were higher in all the groups of children with ASD than in the control group. An alteration in the pattern of cortisol excretion was observed in children with LFA. The level of 5-hydroxyindole acetic acid was higher in children with LFA and MFA than in the control group. On the basis of these findings, the authors stressed that altered cortisol excretion patterns and high level of corticosteroids in urine may be a consequence of altered HPA function, which may eventually contribute to the pathogenesis of ASD. Cortisol diurnal rhythm seems also to be different in ASD children with respect to controls. Tomarken et al. [50] measured salivary cortisol at four time points during the day in a sample of 36 children with ASD and 27 typically developed peers. A decline in evening levels of cortisol was detected, whereas no difference was reported in the morning levels. In particular, 25% of ASD children had an attenuated linear decline in cortisol level, while the trajectory of the other ones was indistinguishable from that of TD children.

Other variables investigated in this field are the diurnal fluctuation of cortisol (DF) and cortisol after awakening response (CAR). Results about DF in ASD population are controversial. Inconsistencies in the reported findings may be related to the differences in age and ASD severity among the investigated samples. In particular, while some studies reported significant differences between ASD patients and controls, others failed to find an association of CAR or DF alterations with ASD [48, 51–54]. Some authors also evaluated if the presence of repetitive behaviors in ASD could be linked to cortisol regulation by measuring diurnal salivary cortisol levels in a sample of ASD children (*n* = 21). Results showed that participants with more severe repetitive behaviors had lower diurnal salivary cortisol than others. As stated by the authors, while repetitive behaviors may be considered a maladaptive strategy for mitigating stress, it is also possible that in this population a down regulation of glucocorticoid system would be associated with prolonged distress [51]. Sharpley et al. [53] specifically focused on ASD females, enrolling 39 girls with high-functioning ASD (diagnosed on the basis of DSM-IV criteria for ASD) for the evaluation of DF and CAR. More than a half of participants showed inverse CAR and more than 14% of the subjects reported inverted DF cortisol concentrations compared to previous models in general population [55]. Three potential sets of predictor factors (physiological, ASD-related, and mood-related) revealed that self-reported depressive symptoms were associated with CAR status, while the most important contributor to the CAR variance was suicidal ideation [53]. Other authors, focusing also on older patients, evaluated it age and puberty may exert a role on cortisol diurnal rhythm in ASD subjects, leading a study a study among ASD individuals of different age ranges (*n* = 113) and controls. Higher evening levels of cortisol, as well as a blunted diurnal slope, were reported in ASD individuals with respect to controls. According to these authors, evening cortisol levels seemed to increase through development in ASD individuals, being higher in adolescents than in children, whereas morning levels of the hormone were generally lower. The authors also suggested that the increase in cortisol level may be involved in the impaired sleep quality [48].

In a more recent work, Baker et al. [7] specifically investigated the relationship between sleep and cortisol levels, comparing ASD adults (*n* = 29) and controls (*n* = 29) by means of questionnaires, actigraphy and salivary samples. ASD participants reported greater reduction in evening cortisol concentrations when compared with controls participants. In the ASD group, poor sleep efficiency and increased wake duration was significantly correlated with cortisol levels measured 1 h before habitual sleep onset time. Moreover, increased sleep onset latency and poorer sleep efficiency was associated with higher subjective arousal in the ASD group. The authors further suggested that low cortisol levels in ASD adults may be linked to a deregulation of HPA axis. During the last years, another study reported alterations in HPA axis in children with ASD, comparing them with subjects with ADHD and typically developed children [54]. A total of 150 children, aged between 6 and 12 years old, were enrolled and distributed into four groups: ADHD, ASD, specific learning disorder (SLP), and typically developed group. Salivary samples were collected at three time points during the day, as well as before and 5 min after an academic performance test and a moral cognition task. ASD children showed lower diurnal salivary alpha-amylase (sAA) secretion, adjusted for age, compared to typically developed ones. Moreover, sAA evening levels resulted significantly higher in ADHD group compared to controls. Authors hypothesized that lower sAA daily output could be related to higher chronic stress in ADHD and ASD children. According to these authors, the academic performance task increased sAA levels in ASD children, while the moral cognition task did not activate the sympathetic nervous system in any group [54].

When considering results from these studies it should be noted that other researches did not find differences between ASD and controls with respect to these parameters. Kidd et al. [56] did not find significant differences between pre-schooling ASD subjects and controls (*n* = 52) with respect to cortisol and sAA levels at different time of the day. However, differences were reported in level variability, which may eventually exert an impact on circadian rhythmicity [56]. Similarly, Corbett et al. [52] comparing CAR in 46 ASD prepuberal males (aged 8–12 years) and 48 controls did not find any significant difference in CAR between the first and the second group. Further studies are needed for clarifying the actual role of HPA-related alterations in ASD (see Table 4).

**Table 4:** Cortisol level and hyperarousal in ASD

References	Participants	Materials	Main findings	Strengths and limitations
Priya Lakshimi et al. [49]	ASD children (*N* = 45 divided into three groups: low, medium and high functioning. Each group included 15 children. *M*/*F* = 36/9, range 4–12 years) Typically developed children (*N* = 45, *M*/*F* = 36/9, range = 4–12 years)	CARS classification as preliminary screening and urinary level of free cortisol, corticosteroids, VMA, and 5-hydroxyindole acetic determination	Corticosteroids excretion levels were higher in all the groups of children with ASD than in the control group. An alteration in the pattern of cortisol excretion was observed in children with LFA. The level of 5-hydroxyindole acetic acid was higher in children with LFA and MFA than in the control group	Limitations: not determined the blood and saliva levels of corticosteroids, free cortisol, VMA, 5-hydroxyindole acetic acid, and prostaglandin E Strengths: group of ASF and controls very identical in number composition, M/F ratio and range age
Gabriels et al. [51]	Pre-pubescent ASD males (*N* = 21, 11 of them with high-RB, and 10 with low RB) Mean age of high-RB group: 7.8 ± 1.7 years Mean age of low-RB: 8.1 ± 1.4 years Range: 3–9 years	Measure of screening: tanner criteria, caregiver-report RBS-R scale, CCIF-RV, SCQ, ADOS, Leiter-R, VABS, BEDS Other measure: salivary cortisol level	Participants with more severe repetitive behaviors had lower diurnal salivary cortisol than others	Limitations: patients were only males and the sample size was low Strengths: patients were carefully selected through different scales
Tomarken et al. [50]	ASD children (*N* = 36, *M* = 30, *F* = 6, Mean age 10.20 ± 1.96) Typically developed controls (*N* = 27, *M* = 23, *F* = 4, mean age = 9.71 ± 1.54) Total range: 7–16 years	Salivary cortisol collection	A decline in evening levels of cortisol was detected, whereas no difference was reported in the morning levels. 25% of ASD children had an attenuated linear decline in cortisol level, while the trajectory of the other ones was indistinguishable from that of TD children	Limitations: disproportioned *M*/*F* ratio in both groups Strengths: controlled study
Sharpley et al. [53]	ASD girls (*N* = 39) Mean age = 10.1 ± 2.7 years Range = 6–17 years	CASI, WASI-II, ADOS-2, DF of cortisol and CAR	Over half of the participants showed inverse CAR and over 14% had inverted DF cortisol concentrations; three potential sets of predictor factors (physiological, ASD-related, and mood) revealed that only self-reported Major Depressive Disorder was significantly associated with CAR status, and that the girls' concern about dying or suicide was the most powerful contributor to the variance in CAR status	Limitations: sample size; statistical power; cultural and geographical isolation; use of a snap-shot design rather than a prospective design; collection of salivary cortisol on a single day; one third of the cohort reported thoughts of “dying or killing oneself; it is not a direct comparison study
Muscatello et al. [48]	ASD youths (*N* = 64, *M* = 57, *F* = 7, mean age 12.02 years) and typically developed youths (*N* = 49, *M* = 42, *F* = 7, mean age = 11.17 years) Total range = 7–17 years	Diagnostic and assessment measures: ADOS, WASI, SCQ, SRS-2, PDS, CBCL, SSS, SES, Salivary cortisol sampling	ASD child, pubertal and adolescents had significantly higher evening cortisol than controls. Adolescent had higher cortisol levels than children	Limitations: predominantly male sample and differences in IQ between groups; the current sample consisted only of those with high-functioning ASD; numeric disproportion between males and females included Strengths: large ASD sample and a comprehensive age range of children and adolescents
Baker et al. [7]	ASD adults (*N* = 29, *M* = 51.7%, *F* = 48.3%, 13 of them were medicated for comorbid anxiety or ASD-Med with mean age = 33.93 ± 6.53 years 16 were drug-free or ASD-only with mean age = 33.55 ± 6.50) and controls (*N* = 29, mean age = 30.99 ± 5.25 years)	Participants completed a questionnaire battery, 14-day sleep/wake diary and 14-day actigraphy assessment; On one day during the data collection period, participants collected five saliva samples, hourly, prior to sleep and two morning samples, immediately upon waking and 30 min thereafter for the analysis of cortisol	ASD participants reported greater reduction in evening cortisol concentrations when compared with controls; In the ASD group, poor sleep efficiency and increased wake duration was significantly correlated with cortisol levels measured 1 h before habitual sleep onset time; increased sleep onset latency and poorer sleep efficiency was associated with higher subjective arousal in the ASD group	Limitations: cortisol was not retained in the model; comorbid diagnoses of anxiety and depression in the ASD-Med group were not confirmed with clinical interviews; small sample sizes Strengths: controlled study with proportioned ASD and controls group; subdivision into ASD group in ASD-Only and ASD-Med
Anesiadou et al. [54]	Four groups: ASD children (*N* = 56, *M* = 49, *F* = 7 mean age = 8.40 ± 1.60 years) ADHD children (*N* = 34, *M* = 22, *F* = 12, mean age = 8.79 ± 1.43 years), SLD children (*N* = 43, *M* = 25, *F* = 18, mean age = 9.55 ± 1.64 years), TD group (*N* = 24, *M* = 16, *F* = 8, mean age = 9.74 ± 1.98 years)	APT, moral cognition task, sAA	ASD children showed lower diurnal sAA secretion, adjusted for age, compared to typically developed ones; sAA evening levels resulted significantly higher in ADHD group compared to controls; the academic performance task increased sAA levels in ASD children, while the moral cognition task did not activate the sympathetic nervous system in any group	Limitations: cross-sectional design does not allow us to make inferences; small sample size of population; sample procedure to a single day; lack of multiple time point sampling; disproportion in *M*/*F* ratio between groups Strengths: four different groups

*ASD* autism spectrum disorder, *CARS* childhood autism rating scale, *LFA* low functioning autism, *MFA* moderately functioning autism, *RBS-R* repetitive behavior scale-revised, *CCIF-RV* child and caregiver information form research version, *SCQ* social communication questionnaire, *ADOS* autism diagnostic observation schedule, *VABS* vineland adaptive behavioral scales, *BEDS* behavioral evaluation of disorders of sleep, *TD* typically developing, *CASI* child and adolescent symptom inventory, *WASI-II;* *ADOS-2* Wechsler abbreviated scale of intelligence second edition, *DF* diurnal fluctuation, *CAR* cortisol awakening response, *SRS-2 *social responsiveness scale second edition, *PDS* pubertal development scale, *IQ* intelligence quotient, *CBCL* child behavior checklist, *SSS* stress survey schedule, *SES* socio-economic status, *ASD-Med* comorbid diagnoses of anxiety and depression, *APT* academic performance test, *sAA* salivary alpha amylase

## Autonomic nervous system (ANS) alterations in ASD

Several studies highlighted altered ANS responses in ASD, which may be related to altered circadian rhythms. Alteration of autonomic variables in ASD was reported since early infancy [57]. Noticeably, alterations of ANS have also been implicated in metabolic, sleep, gastrointestinal disorders, as well as in seizures and hormonal dysfunction [58]. On the other hand, the association between autonomic instability and psychiatric symptoms like depression, anxiety and sleep disorders was previously stressed in the literature [57, 58].

In the field of ASD, differences in heart rate (HR) response between ASD children and controls were frequently highlighted, and both HR and respiratory sinus arrhythmia (RSA), which may be a good index of CNS regulation of HR, have been used as indicators of the integrity of the ANS [59]. In this framework, a recent study, evaluating HR and RSA among subjects from 1 to 72 months of age at 8 different time points, found a progressive reduction in HR and increases in RSA. However, the RSA increase was of smaller entity among those subjects who later received a diagnosis of ASD [59]. Bharath et al. [8] evaluated in a sample of ASD children (*n* = 40) and controls (*n* = 40) HRV together with urinary levels of VMA. They found that low frequency HRV was more represented in the ASD group, while high frequency HRV was less represented. In addition, urinary VMA concentrations were higher among ASD children. The authors hypothesized that ASD children may have a reduced cardio-vagal activity as measured by HRV, and increased sympathetic activity as measured by urinary VMA. Another study in a smaller sample further highlighted how HR variability (HRV) might be a promising parameter for differentiating ASD children from controls also in a very early stage. The authors enrolled 20 children (6 children with ASD, 14 controls), recording photo plethysmography (PPG) for 4 min in each child during resting state and color stimulus test condition. PPG was used for deriving HRV. Different responses between ASD and controls during resting state were highlighted for specific features of HRV [60]. Thapa et al. [61] compared ASD adults (*n* = 55) and controls from the general population (*n* = 55), and reported a significantly higher resting state HR among ASD patients, together with lower square root of mean squared differences of successive R–R intervals (RMSSD) and lower high frequency HRV. No significant difference was reported for low frequency HRV. These authors hypothesized that their results may suggest a general deregulation in resting autonomic activity among ASD adults, characterized by lower parasympathetic activity.

Among other factors linked to ANS alterations, pupil size was also considered as a potential biomarker for ASD. Anderson et al. [62] compared a group of ASD children with age matched controls, reporting that ASD individuals seemed to show a larger tonic pupil size and lower afternoon levels of sAA. The authors also highlighted a little diurnal variation of sAA among ASD children, while among controls a linear increase throughout the day was observed. Globally, they hypothesized that these features may be associated with an over-production of Norepinefrine (NE) [62]. In a recent work, Arora et al. [63] reviewed previous studies that evaluated autonomic arousal by measuring HR, pupillometry and electrodermal activity (EDA) in ASD individuals compared with controls. A general hyperarousal in ASD individuals with respect to controls was reported during resting states, eventually linked to the difficulties showed by ASD people in regulating their arousal.

Considering the issue of sleep problems, some studies specifically focused on the relationship between ANS and sleep alterations in ASD. In particular, Pace et al. [64] investigated HRV during sleep in high-functioning ASD children (*n* = 19) and controls (*n* = 19). Two indices of cardiac activity, the high frequency (HF) spectrum and RMSSD, were significantly higher in ASD individuals when compared with other ones. In both groups, lower mean HR values were found during sleep with respect to those registered during wakefulness. However, the ASD group showed a lower decrease in HR during deep sleep despite the presence of a higher parasympathetic tone. Another study analyzed HR and HRV during sleep in ASD. A group of ASD children (*n* = 21) and controls (*n* = 23) was evaluated by means of overnight polysomnography. HR was found higher in ASD subjects than in controls during stages N2 and N3 of non-REM sleep and during REM sleep. High frequency oscillations of HRV, which reflect vagal modulation, resulted lower in N3 stage of non-REM sleep and in REM sleep among ASD children. Low frequency to high frequency ratio (LF/HF) was instead higher during REM sleep. These data, together with the higher HR, lead to hypothesize a sympathetic dominance during sleep among ASD children, which would be associated with a decreased vagal influence [65]. Tessier et al. [66] investigated HRV in ASD subjects (17 adults and 13 children) and typically developed ones (16 adults and 13 children) before and after sleep. They divided the sample into four groups: ASD and control adults and ASD and control children. Each group was evaluated for two nights in a sleep laboratory. Normalized values of low frequency, normalized values of high frequency and LF/HF ratio were evaluated during sleep and vigilance state. Morning high frequency values resulted to be lower among ASD adults when compared with controls. LF/HF ratio during REM sleep was instead higher in both ASD adults and controls than in children. These results are in line with a lower parasympathetic activity in ASD individuals in the morning. However, it should be noted that while this study provided a good insight on possible differences in the investigated parameters depending on age, the presence of multiple group comparison analyses in a limited sample size limited the extensibility of their results. More recently, Chong et al. [67] investigated the relationship between EDA, another parameter used as a marker of sympathetic nervous system activation, and sleep deregulation in ASD children (*n* = 13). Two EDA indices, non-specific skin conductance responses (NSSCR) and tonic skin conductance levels (SCL) were measured. The group of children with deregulated sleep showed fewer NSSCRs and lower SCL in the afternoon.

Finally, other confirmations of the presence of altered ANS response in ASD may came from pharmacological studies: some authors proposed propranolol, a beta-blocker drug, as a possible therapeutic option for dysautonomias and emotional behaviors in ASD. According to the available literature, this drug seems to significantly improve cognitive performances, such as verbal problem solving, social skills, mouth fixation and conversation reciprocity. Moreover, it seems to improve anxiety, behavioral and autonomic deregulation, aggressive, self-injurious, and hypersexual behaviors, including among subjects with acquired brain injury. However, the specific benefits with respect to sleep still need to be further investigated [68].

The above described literature seems to suggest the presence of hyperarousal states in ASD individuals, possibly with a hyper-sympathetic state not properly counterbalanced by the vagal parasympathetic influences [62–67]. However, also in this case the available studies are still scant in number and highly heterogeneous in methodology. Moreover, studies specifically focused on the possible relationship between altered ANS function and sleep alteration are even fewer and did not allow reaching a conclusive agreement on this topic (see Table 5).

**Table 5:** Autonomic nervous system deregulation in ASD

References	Participants	Materials	Main findings	Strengths and limitations
Anderson et al. [62]	Study 1: ASD group (*N* = 12, *M* = 11, *F* = 1, mean age = 50.25 months, range = 30–69 months), down syndrome (DS) group (*N* = 9, *M* = 7, *F* = 2, mean age = 48.67 months, range = 20–73 months), TD group (*N* = 11, *M* = 10, *F* = 1, mean age = 51.73 months, range = 34–69 months) Study 2: ASD group (*N* = 18, mean age = 57.78 months, range = 39–73 months), TD group (*N* = 19, mean age = 52.26 months, range = 33–79 months)	Tonic pupil size, saliva sample collection	ASD showed larger pupil size and lower sAA levels than controls; sAA was strongly correlated with tonic pupil size; typical controls showed a linear increase in sAA during the day	Limitations: small sample size, disproportioned M/F ratio in groups of the first study, not stratification in the second study for males and females Strengths: two different studies, presence of two a control groups (healthy individuals and down syndrome)
Pace et al. [64]	ASD group (*N* = 19, mean age = 10.7 ± 1.2 years), control group (*N* = 19, mean age = 9.9 ± 1.6 years)	Questionnaire, actigraphy; nocturnal recordings; HRV analysis	Lower mean HR values were found during sleep with respect to those registered during wakefulness; however, the ASD group showed a lower decrease in HR during deep sleep despite the presence of a higher parasympathetic tone	Limitations: small sample size, not stratification in males and females Strengths: presence of a control group
Harder et al. [65]	ASD children (*N* = 21, all males, mean age = 7.8 ± 1.8 years) and typically developed children (*N* = 23, *M* = 18, *F* = 5, mean age = 8.0 ± 1.9 years)	Polysomnography, HR and HRV	In both groups, HR decreased during non-REM sleep and increased during REM sleep; HR was significantly higher in stages N2, N3 and REM sleep in the ASD group; ASD children showed less HF modulation during N3 and REM sleep; LF/HF ratio was higher during REM; heart rate decreases with age at the same level in ASD and in TD. LF was influenced by age	Limitations: small sample size, ASD children were composed only by males Strengths: controlled study
Tessier et al. [66]	ASD children (*N* = 13, range 7–12 years, mean age = 10.2 ± 2.1), ASD adults (*N* = 16, range = 16–27 years, mean age = 22.0 ± 3.8 years), TD children (*N* = 13, range = 6–13 years, mean age = 10.5 ± 1.8 years), TD adults (*N* = 17, range = 16–27 years, mean age = 21.1 ± 4.0 years)	Sleep laboratory measures, ECG recordings	Results show that ASD adults had lower HFnu in the morning than TD adults. During REM sleep, adults had higher LF/HF ratio than children, regardless of their clinical status	Limitations: high number of males, ASD participants were medicine-free; LH/FH ratio significance has been largely questioned Strengths: four different equally subdivided groups (children and adults with or without ASD)
Bharath et al. [8]	ASD children (*N* = 40, *M* = 24, F = 16, range = 5.25–12 years, mean age = 10 years), TD controls (*N* = 40, *M* = 26, *F* = 14, range = 7.25–11.75 years, mean age = 9 years)	Autonomic index was assessed by the analysis of short term HRV; urinary levels of VMA estimation was used as a biochemical autonomic index	ASD children exhibit lower cardio-vagal activity as measured by HRV and increased sympathetic activity as assessed by urinary VMA compared to that of TD children	Limitations: small sample size, difference in *M*/*F* ratio Strengths: presence of a control group similar to ASD ones (same number of participants)
Sheinkopf et al. [59]	Infants later diagnosed with ASD (*N* = 12, *M* = 12, *F* = 0) and controls non-later ASD (*N* = 106, *M* = 58, *F* = 48) range: 1–72 months	HR and RSA	Both groups showed an expected age-related decrease in HR and increase in RSA, without difference in rate of HR decrease over time; ASD infants demonstrated a smaller linear increase in RSA, indicating slower growth in RSA over time in comparison to controls, thus suggesting that differences in physiological regulation may develop with age in ASD	Limitations: small sample size; participants were drawn from a high-risk cohort designed to investigate the developmental effects of prenatal drug exposure, which could have effects on RSA at one moth of age; disproportion between two groups composition Strengths: controlled study
Thapa et al. [61]	ASD group (*N* = 55, *M* = 74.5%, *F* = 25.5%, mean age = 23.11 ± 5.98 years) control group (*N* = 55, *M* = 80%, *F* = 20%, mean age = 22.00 ± 5.24 years)	HRV	Difference in resting-state HRV between adults diagnosed with ASD compared to the neurotypical control group, with lower parasympathetic activity in ASD	Limitations: ASD group had psychiatric comorbidities, whose effect was difficult to determine due to small sample size; two different devices were used to determine HRV; majority of patients were males Strengths: presence of a control group
Mohd et al. [60]	ASD children (*N* = 6), TD controls (*N* = 14)	HRV derived from PPG	HRV response can differentiate between ASD and TD children and could contribute to the detection of ASD to facilitate the children getting the best intervention at the earliest possible time	Strengths: controlled study Limitations: small sample size, not stratification in age and sex
Chong et al. [67]	ASD children (*N* = 13) divided in: dysregulated sleep group (*N* = 7, *M* = 63%, *F* = 37%, mean age = 7.53 ± 1.35 years) Regulate sleep group (*N* = 6, *M* = 80%, *F* = 20%, mean age = 4.46 ± 1.28 years)	Actigraphy for sleep measure, EDA (which included NSSCR and SCL, SCQ for ASD symptoms core, VABS-II for adaptive behavior	Children in the dysregulated sleep group had fewer NSSCRs and lower SCL in the afternoon	Limitations: small and heterogeneous sample; prevalence of males in both groups; absence of a control group Strengths: subdivision in two groups based on facility/difficulty in sleeping

*ASD* autism spectrum disorder, *sAA* salivary alpha amylase, *HR* heart rate, *HRV* heart rate variation, *REM* rapid eye movement, *HF* high frequency, *VMA* vanillylmandelic acid, *TD* typically developing, *RSA* respiratory sinus arrhythmia, *PPG* photoplethysmography, *EDA* electrodermal activity, *NSSCR* non-specific skin conductance responses, *SCL* skin conductance levels, *SCQ* social communication questionnaire, *VABS-II* vineland adaptive behavior scale second edition

## Current pharmacological perspectives for sleep disorders in ASD

To date, the first line treatment for ASD sleep problems, especially in children, are sleep hygiene practices and behavioral interventions [34, 69]. There are some extinction measures which parents of ASD children with sleep problems may follow, such as decreasing afternoon naps, or placing the child in his/her own bed without extraneous stimuli [70]. Associated behavioral manifestations, such as aggressive behaviors, may also be controlled by atypical antipsychotic agents such as risperidone or aripiprazole [71].

Among sleep related pharmacological interventions, administration of melatonin and its agonist drug agomelatine was reported to reduce latency of sleep and the number of awakenings in ASD children. As reported in the previous chapters, in ASD populations a reduction or an alteration in melatonin secretion was highlighted, as well as a reduction in gamma-Aminobutyric acid (GABA)_B_ receptors in the anterior and posterior cingulate cortex and in the fusiform gyrus cortex [12].

In this framework, Wasdell et al. [72] led a study on 51 children with neurodevelopmental disabilities (age range 2–18 years), who were administered melatonin 20–30 min before the desired bedtime and were invited not to eat during the 2–3 h before the moment of drug assumption. Melatonin resulted to be effective in 47/51 children in improving sleep quality and reducing family stress. Malow et al. [73] performed instead a dose escalation study on 24 children with ASD, who were free of psychotropic medications. The dose response, tolerability and safety were studied during a 14-week open label design. The supplementation of melatonin (1–3 g) showed to improve sleep latency and was proved to be tolerated and safe. A work by Maras et al. [74] has proved 3-month efficacy and safety of a novel pediatric-appropriate prolonged-release melatonin (PedPRM), an easily swallowed formulation shown to be efficacious versus placebo, for long-term treatment (that is up to 52 weeks) of children with ASD who suffered from insomnia. The study also reported an improvement in caregivers’ quality of life. While several studies were conducted in this field, also review and meta-analyses are available: in particular, a meta-synthesis by Cuomo et al. [75], collected data from eight previous systematic reviews based on a total of 38 studies about sleep intervention in ASD. Interventions were categorized in four groups: melatonin supplementation, other pharmacological therapies (such as risperidone, clonidine, benzodiazepines), behavioral interventions, parent education/education programs, alternative therapies. Melatonin supplementation was showed to be the most successful intervention for sleep initiation and maintenance of sleep, while for other parameters, such as night wakings or self-settlings, a strong effect of behavioral intervention and education was reported. Despite the general agreement on the efficacy of melatonin for improving sleep parameters and daytime behaviors in ASD [36], it should be noted that studies which evaluated melatonin efficacy are affected by several limitations, such as small sample sizes, comorbid neurodevelopmental disabilities, heterogeneity in methodologies and in dosages, and that not all the studies clearly confirmed melatonin beneficial effects on all the outcomes [76]. Adverse effects linked to melatonin use were also reported, although generally mild, such as morning tiredness, headache, irritability, diarrhea or night-time awakenings [35, 36].

More limited evidence is available for other pharmacological interventions. Ramelteon, a melatonin agonist and a sleep-cycle regulator used for people who have difficulties in falling asleep, was also hypothesized to have a preventive effect on the onset of sleep disorder after general anesthesia in patients with ASD [77]. A case series is also available, which reported, during a trial on 3 ASD children with Ramalteon, a decrease in sleep problems such as insomnia as well as an improvement of ASD behavioral symptoms [78, 79]. The use of agomelatine was also evaluated in this population. Ballester et al. [80] highlighted among ASD subjects (*n* = 23) with sleep problems a significant increase of night total sleep time, together with phase correction and improvement of sleep stability, after 3-months of treatment with agomelatine. Other studies reported that donepezil, a reversible inhibitor of acetyl cholinesterase, may have beneficial effects on ASD individuals for improving sleep, communication, eye contact, hyperactivity, expressive and receptive speech [81, 82]. Donepezil combined with choline supplement also showed a sustainable effect on language skills in children with ASD for 6 months after treatment, particularly in subject aged below 10 years [83]. Finally, benzodiazepines, which are commonly used in other neuropsychiatric conditions, were also reported to improve some sleep problems in ASD, even if paradoxical reactions with agitation and hyperactivity were reported [84, 85]. The use of benzodiazepines in ASD might be supported by evidences of a GABA-A alpha 5 receptor deficit in this population [85], while an impairment in GABAergic functions were also highlighted in ASD murine models [86–88]. In a case series of 11 ASD children, the administration of 0.5–1 mg of clonazepam showed efficacy in 75% of the subjects [84]. However, the possibility that children with neurodisabilities could show an increased risk of paradoxical reaction prevents many clinicians from prescribing benzodiazepines in ASD children [88]. Another drug which has been object of investigation in this field is clonidine. In a case series of six children with neurodevelopmental disorders, clonidine was proven effective for reducing sleep problems and sleep initiation latency but showed a small impact on recurrent night time and early morning awakenings, mood instability and aggressiveness [89]. Among antidepressants, trazodone was suggested to be useful for treating the advanced sleep phase in ASD individuals [90]. Mirtazapine was reported to be effective on insomnia and symptoms related to sleep deprivation such as irritability and anxiety. In a study led by Posey et al. [91], 25 ASD participants were investigated to test the efficacy and tolerability of mirtazapine. An improvement of several symptoms, including aggressive behaviors, self-injury and irritability, was observed in nine subjects. A further research analyzed the effects of gabapentin administration to ASD children (*n* = 23). Gabapentin administered 30–45 min before the bedtime resulted to be effective in improving sleep in 18 participants [92, 93]. On the basis of the lower ferritin levels frequently reported among ASD children, an 8-week open-label treatment with oral iron supplementation was also conducted in 33 ASD children with restless sleep. A significant improvement in sleep quality was reported [11, 94].

Globally, it should be noted that while melatonin studies should be regarded in light of the previously mentioned limitations, it appears clear how studies on pharmacological interventions besides melatonin are still very limited in number, featuring also small sample sizes or, for some drugs, being limited to case series, thus increasing the risk of biases and placebo effects. As a result, the above reported research should be considered cautiously until confirmed by further investigations. In addition, another issue in this field lies in the fact that in most of the studies, including those on melatonin, the pharmacological treatment was reported to improve also other symptoms besides sleep problems. Although these data may further support the beneficial effect of the treatment object of investigation, it may be also considered a confounding factor: further studies should clarify if these drugs would exert a specific effect on sleep, or the sleep improvement should be considered a consequence of the improvement of other symptoms (see Table 6).

**Table 6:** Pharmacological therapies for sleep problems in ASD

References	Participants	Materials	Main findings	Strengths and limitations
Posey et al. [91]	Subjects with neurodevelopmental disorder (PDDs), (*N* = 26, *M* = 21, *F* = 5, range = 3.8–23.5 years, mean age = 10.1 ± 4.8 years, 20 of them with ASD, 1 with Asperger’s disease, 1 with Rett’s disorder, 4 with PDDs not specified)	Treatment with mirtazapine (dose range = 7.5–45 mg daily, mean = 30.3 ± 12.6 mg daily)	Mirtazapine did not improve core symptoms of social or communication impairment. Adverse effects were minimal and included increased appetite, irritability, and transient sedation	Limitations: lack of a control group, disproportion between male and female number in group, small sample size
Thirumalai et al. [84]	ASD patients (*N* = 11, range 3–9 years, *M* = 9, *F* = 2, mean age = 5.09 years)	Polysomnography, EEG, EMG	REM sleep behavior disorder was identified in 5 of these 11 patients. Since REM sleep behavior disorder typically affects elderly males with neurodegenerative diseases, the identification of this phenomenon in autistic children could have profound implications for our understanding of the neurochemical and neurophysiologic bases of autism. Accurate diagnosis of REM sleep behavior disorder would enable specific treatment with clonazepam and help the family and the child	Limitations: small sample size, absence of a control group, disproportion between males and females
Ingrassia et al. [89]	Children (*N* = 6, 3 ADHD and 3 with mental retardation, range = 6–14 years, mean age = 11.2 years)	Clonidine administration (range dose = 50–100 mcg daily)	All children showed maintained improvements in their sleep pattern following the use of clonidine with only mild side-effects reported	Limitations: small sample size, lack of a control group, case series
Dosman et al. [94]	ASD children (*N* = 33, *M* = 27, *F* = 6, mean age = 6 years and 6 moths, range = 2 year 8 months–10 year 8 months)	Questionnaires (Sleep Disturbance Scale for Children, movements during sleep scale of Chervin and Hedger, Food records) made by parents after iron supplementation	High prevalence of restless sleep, which improved with oral iron supplementation, suggests that sleep disturbance may be related to iron deficiency in autism	Limitations: absence of a control group, disproportion between males and females
Rossignol et al. [36]	Initial studies review: 35 reviewed independently by two reviewers Five of them were investigated through meta-analysis	Database used: PubMed, Google Scholar, CINAHL, EMBASE, Scopus, ERIC Quality of studies was assessed through Downs and Black checklist	Six studies reported improvements in daytime behavior using melatonin; 18 studies on melatonin treatment in ASD reported improvements in sleep duration, sleep onset latency, night-time awakenings From the meta-analysis: improvements in sleep duration but not in night-time awakenings	Strengths: the meta-analysis increases the statistical significance, funner plot didn’t indicate publication bias Limitations: small sample size, protocol which measured changes in sleep parameters were variable
Buckley et al. [81]	ASD subjects (*N* = 5, range = 2.5–6.9 years) compared with within-lab controls	Polysomnography for REM sleep augmentation after donepezil administration	REM sleep as a percentage of Total Sleep Time was increased significantly and REM latency was decreased significantly after drug administration in all subjects	Limitations: open-label study without controls; very small sample size
Malow et al. [73]	ASD children (*N* = 24, range = 3–9 years, mean age = 5.9 years)	Melatonin supplementation, Actigraphy, Children’s Sleep Habits Questionnaire (CSHQ), Child Behavior Checklist (CBCL) scale	Supplemental melatonin improved sleep latency, as measured by actigraphy, in most children at 1 or 3 mg dosages. It was effective in week 1 of treatment, maintained effects over several months, was well tolerated and safe, and showed improvement in sleep, behavior, and parenting stress	Limitations: absence of a control group, small sample size
Mendez et al. [85]	ASD people (*N* = 3, range = 34–43 years, mean age = 39.33 years), controls (*N* = 3, range = 37–40 years, mean age = 38.66 years)	PET with receptor PET ligand [11C] Ro15-4513 was used to measure a1 and a5 subtypes of the GABA-A receptor levels	Lower [11C]Ro15-4513 binding was found throughout the brain of participants with ASD compared with controls. Planned region of interest analyses also revealed significant reductions in two limbic brain regions, namely the amygdala and nucleus accumbens bilaterally, thus suggesting a GABA-A a5 deficit in ASD	Limitations: very small number of participants Strengths: controlled study, accurate technique of investigation (PET)
Maras et al. [74]	Initial participants: Children (*N* = 125, 96,8% of them ASD, 3,2% with Smith-Magenis syndrome, range = 2–17.5 years) Final number of participants: *N *= 95, 51 of them received PedPRM, 44 placebo	Administration of 2, 5, or 10 mg PedPRM; Measures were: CSDI, PSQI, ESS, quality of life WHO-5 Well-Being Index	PedPRM, an easily swallowed formulation shown to be efficacious versus placebo, is an efficacious and safe option for long-term treatment (up to 52 weeks reported here) of children with ASD and NGD who suffer from insomnia and subsequently improves caregivers' quality of life	Limitations: open-label design of the study; lack of a control group made by healthy individuals; some individuals discontinuated treatment Strengths: presence of a group receiving placebo
Ballester et al. [80]	ASD people (*N* = 23, *M* = 83%, mean age = 35 ± 12 years)	Administration of agomelatine or placebo	Agomelatine was effective and well tolerated for treating insomnia and circadian rhythm sleep problems present in adults with ASD and ID	Limitations: small sample size; absence of a control group Strengths: placebo-controlled study
Gabis et al. [83]	ASD children (*N* = 60, range = 5–16 years, mean age = 9.5 ± 3.22 years)	AChE inhibitors and choline supplements in children and adolescents with ASD	Combined treatment of donepezil hydrochloride with choline supplement demonstrates a sustainable effect on receptive language skills in children with ASD for 6 months after treatment, with a more significant effect in those under the age of 10 years	Limitations: safety concerns limited the dose and the compounds used in the study; small sample size; two different language tests were used to assess global language skill; absence of a control group

*ASD* autism spectrum disorder, *PDDs* pervasive developmental disorders, *EEG* electroencephalography, *EMG* electromyography, *ADHD* attention deficit hyperactivity disorder, *C-GIS* clinical global impression severity, *PGAS* parents global assessment scale, *CSHQ* children’s sleep habits questionnaire, *CBCL* child behavior checklist, *PET* positron emission tomography, *GABA* gamma-aminobutyric acid, *PedPRM* pediatric-appropriate prolonged-release melatonin, *CSDI* composite sleep disturbance Index, *PSQI* Pittsburgh Sleep Quality Index, *ESS* Epworth sleepiness scale, *NGD* neurogenetic disorder, *AChE* acetylcholinesterase

## Biological correlates of altered circadian rhythm and sleep problems in ASD: broadening the perspective

Studies on sleep showed how ASD subjects typically report more problems regarding bedtime resistance and reduced sleep pressure [16]. A link between sleep difficulties and irritability, deficits in social skills and behavioral problems was also highlighted in ASD children [13, 14, 19]. Generally, an insufficient sleep time seems to affect the quality of life of ASD individuals [23], including an important impact on employment status [22]. As reported in the previous chapters, several mechanisms may be responsible of this feature. The molecular explanation of circadian problems in ASD individuals may be found in mutation, variants or different expression of specific genes which regulate circadian rhythms, such as *CLOCK* [5, 26, 29], *PER1* [27] and CACNA1C [30]. On the other hand, these problems may also be related to an alteration of melatonin levels (and, eventually, of PGV) as reported by several studies [3, 6, 32, 44, 45]. In addition, an impaired circadian regulation of cortisol [48, 56], as well as increased urinary corticosteroids levels were reported in ASD, possibly due to an altered HPA axis function [49]. Besides the development of anxiety and depressive symptoms, the chronic activation of HPA axis seems to exert a negative impact on ASD individuals’ daily life [48, 95] and to inversely correlate with gravity of repetitive behaviors [51]. Studies focused on autonomic functions in ASD globally pointed out the presence of hyperarousal state in ASD individuals [63]. ASD was hypothesized to be associated with the presence of a hyper-sympathetic state, which would be not adequately compensated by vagal parasympathetic influences. The analysis of autonomic indices such as pupil size [62], electrodermal activity [67] and HR highlighted a tendency towards a lower parasympathetic activity during daytime and a sympathetic dominance during sleep [64–66].

It should be taken into account that some of the above reported alterations in ASD were not associated only with sleep problems but also with more ASD-specific clusters of symptoms, such as communication impairment or repetitive behaviors [51, 96]. These data suggest that the altered sleep patterns in ASD should be regarded in a broader perspective, considering the possible bidirectional interactions between ASD core symptoms and sleep problems in promoting and maintaining each other. Noticeably, the importance of rhythmicity and synchrony of motor, emotional and interpersonal rhythms for the development of social communication was recently stressed [97]. In addition, ASD children were reported to have difficulties in adapting their changes to the internal or external environment [98]. It would be worth mentioning that epileptic seizures, which are often comorbid with ASD, may be influenced by rapid rhythms of sensory stimuli in the external environment [99]. It was also hypothesized that melatonin may be implicated in the synchronization of the circadian clock network. As a result, interventions which combine melatonin treatment with behavioral measures may be useful for investigating the alterations of this clock [98]. The contribution of melatonin in the ontogenetic establishment of circadian rhythms and in the synchronization of circadian clocks suggests that melatonin may also be implicated in motor, emotional and interpersonal rhythms. An altered melatonin excretion or activity has been related to both timing problems in biological clocks and to the severity of social communication impairment [96, 100, 101]. Maternal variations in cardiac rhythm and in hormone levels were reported to influence the child’s ability to adjust to the environment, while parent–infant synchrony and the construction of shared timing were supposed to be important for social communication development [102]. Some authors also hypothesized that repetitive behaviors may be interpreted as a coping strategy for controlling anxiety and stress response as well as for experiencing more continuity and stabilizing circadian rhythms [51, 103]. In this framework, it should be remembered that melatonin seems to exert an effect also on the improvement of stereotyped behaviors [73]. Both melatonin supplementation and Early Start Denver Model (ESDM) have been studied in ASD not only for sleep problems but also for improving social communication, stereotyped behavior, rigidity, and anxiety. A significant improvement in global communication (including verbal and non-verbal scores) was reported [10], while improvements in rigidity were highlighted on the basis of parents’ and teachers’ comments [104]. Wasdell et al. [72] led an investigation to test the efficacy of melatonin in treatment of delayed sleep phase syndrome and sleep maintenance problems in children with neurodevelopmental disabilities and ASD, highlighting an anxiety reduction based on caregivers’ comment. ANS dysfunctions were also hypothesized to be involved in different ASD symptoms. Condy et al. [9] reported that repetitive behavior severity seems to be predicted by base line RSA and by its reactivity. Low baseline cardiac vagal control predicted less adaptive behaviors in children with and without ASD. ANS studies which analyzed HRV, pupil size and sAA, highlighted that ASD subjects differ from typically developed ones, showing a general reduction in parasympathetic function, while a tendency towards hypoarousal was reported in ASD by studies which measured electrodermal responsiveness [63]. Kaartinen et al. [105] investigated the relationship between autonomic arousal to direct gaze (measured by means of skin conductance responses) and social impairment in ASD children (*n* = 15) and controls (*n* = 16). Among ASD children, but not among controls, a positive association was found between impairment in social skills and increased arousal enhancement to direct gaze. In another study, RSA and HR were instead correlated with the accuracy and latency of recognition of facial emotions. Children with ASD were slower in recognizing facial emotions and made more errors in recognizing anger. While ASD subjects showed lower amplitude RSA and higher HR than controls, ASD children with more amplitude of RSA were faster at recognizing emotions. It was proposed that ASD children may live in a hyper-sympathetic state with a reduced ability to calm down. This asset may contribute to their impaired ability to engage in social interactions and to the increased levels of anxiety [106]. Bazelmans et al. [107] highlighted significant correlations between language skills and HR in non-ASD children and between language skills and HRV in ASD ones. These authors also stressed the possible use of autonomic control for detecting individual differences among ASD subjects. On the other hand, another study did not find specific associations between social functioning and autonomic/cortisol response in ASD and non-ASD subjects [108]. Recently, the role of gut microbiota in ASD physiopathology is also acquiring more attention. In particular, gut microbiota and CNS were supposed to influence each other through the “gut brain axis”, which would also involve mucosal immune system, enteric and autonomic nervous system (in particular the vagal branch) [109, 110]. In this framework, a recent investigation further broadened the perspective, stressing the importance of evaluating both microbiota and ANS alterations to properly differentiate specific ASD presentations. The authors evaluated autonomic functions and microbiota composition among ASD individuals comparing them with their non-affected first-degree relatives. A lower interbeat interval was reported in ASD patients, which might be eventually associated with higher sympathetic arousal and lower vagal tone. ASD individuals with sleep disturbances showed more gastrointestinal dysfunctions as well as altered interbeat interval and blood volume pulse, suggesting a possible role of these indices for detecting occult sleep disturbances among ASD subjects. Moreover, ASD subjects with greater microbial richness (increased alpha diversity of the gut flora) reported milder sensory/cognitive and language/communication impairment [110]. However, it should be noted that the use of first-degree relatives as controls should be considered as a major limitation for this study, considering the increasing interest about sub-threshold forms of autism [111–113]. Increasing literature is pointing out that autistic traits would be distributed in a continuum in the general population, being highly represented in clinical populations of psychiatric patients with other disorders as well as in first-degree relatives of ASD probands [114–118]. In this latter population, the presence of sub-threshold autistic traits also contributed in shaping the concept of “broad autism phenotype”. In this framework, biological studies in ASD field should include the category of patients’ relatives as an intermediate group and not as a control group [113, 118].

## Conclusions

Globally, literature about the issue of sleep disturbance in ASD did not reach a sufficient agreement, especially when considering possible biochemical correlates. Further studies should investigate the link between ASD and sleep disturbances in a broader perspective, which would allow evaluating the shared biological underpinnings between ASD symptomatology and altered circadian rhythms. Moreover, an integrative model would allow clarifying the possible bidirectional interaction between sleep problems and other ASD symptoms: while disrupted sleep may worsen ASD clinical picture, exerting a detrimental effect on cognitive and behavioral features, on the other hand the typical core of ASD symptoms may facilitate the presence and maintenance of sleep problems in this population. In addition, pharmacological studies for sleep problems in ASD need to follow more standardized protocols to reach more repeatable and reliable results. As circadian rhythm alterations are known to exert a detrimental effect on psychopathological conditions, including ASD, improving sleep patterns in ASD subjects may lead to a significant improvement of the clinical outcome and of the psychopathological trajectory, eventually reducing also the risk of developing further disorders in comorbidity [47, 114]. Improve our knowledge in this field may lead to detect potential pathognomonic alterations or even biological markers for ASD, with a consequent improvement of diagnostic strategies. On the other hand, a better understanding of biological bases of ASD symptoms may pave the way for the investigation of further therapeutic targets, eventually improving actual treatment strategies for a population which is still poorly responsive to the currently approved treatments.

## Data Availability

Not applicable.
